# PF4 inhibits ferroptosis-mediated intracerebral hemorrhage through modulating the CXCR3/AKT1/SLC7A11 signaling pathway

**DOI:** 10.17305/bb.2024.11283

**Published:** 2024-11-19

**Authors:** Na Hu, Guohong Zhang, Liping An, Wei Wang, Ran An, Yunfeng Li

**Affiliations:** 1 Department of Biochemistry and Biology, School of Pharmacy, Hebei University of Chinese Medicine, Shijiazhuang, Hebei Province, China; 2 Hebei Key Laboratory of Chinese Medicine Research on Cardio-Cerebrovascular Disease, Shijiazhuang, Hebei Province, China; 3 The Seventh People's Hospital of Hebei Province, China

**Keywords:** Platelet factor 4, PF4, intracerebral hemorrhage, ICH, ferroptosis, CXCR3/AKT1/SLC7A11 pathway, inflammatory response

## Abstract

Ferroptosis plays a crucial role in the secondary pathophysiological damage to brain tissue surrounding hematomas after intracerebral hemorrhage (ICH). While platelet factor 4 (PF4) is known to promote regeneration following peripheral nerve injury, its role in brain tissue repair after cerebral hemorrhage remains unclear. In this study, hemin-induced PC12 cells were treated with various inhibitors and assessed for viability, oxidative stress, and ferroptosis using a combination of assays, including CCK-8 (Cell Counting Kit-8), EdU (5-ethynyl-2’-deoxyuridine), flow cytometry, and immunofluorescence. ICH cells were also treated with recombinant PF4 (Rm-PF4) and a CXCR3 antagonist (AMG487) to investigate the mechanism by which Rm-PF4 influences hemin-induced PC12 cell injury and inflammation. Subsequently, ICH mouse models were established via collagenase injection. Neurological function in these mice was evaluated using the Cylinder and Corner tests. Histopathological damage to brain tissue was analyzed through HE, TUNEL, and Nissl staining, as well as immunohistochemistry (IHC), to further explore the role of Rm-PF4 in controlling neuroinflammation in vivo. Results showed that Rm-PF4 inhibited hemin-mediated ferroptosis-induced PC12 cell damage and inflammation by activating the CXCR3/AKT1/SLC7A11 signaling pathway. Blocking the CXCR3/AKT1/SLC7A11 pathway partially reversed PF4’s protective effects on hemin-induced PC12 cells. In ICH mice, pro-inflammatory marker CD16 (3rd day) and anti-inflammatory marker Arg1 (7th day) were significantly decreased and increased, respectively (*P* < 0.05). IL-6, TNF-α, and IL-1β levels were downregulated in brain tissues after Rm-PF4 injection, which was significantly reversed by AMG487. PF4 inhibits ferroptosis after ICH reduced PC12 cell damage and the inflammatory response via activating the CXCR3/AKT1/SLC7A11 pathway.

## Introduction

As a country with a high incidence of hemorrhagic stroke, our citizens face a lifetime risk of up to 15.5%, which is increasing at a rate of 1.7% per year [1, 2]. Intracerebral hemorrhage (ICH) is the most prevalent type of hemorrhagic stroke and is one of the disorders with the highest morbidity, disability, and fatality rates, affecting the lives and health of millions of people globally each year [3]. Approximately 35%–52% of ICH patients die within 30 days of the commencement of the disease, and only 20% of patients can attain self-care within six months after the onset of the disease, creating a tremendous economic burden on the state, society, and many families [1, 4, 5]. Brain injuries caused by ICH are mainly divided into primary and secondary brain injuries. Primary brain injury is mainly caused by the mechanical compression effect of hematoma, while the pathological process of secondary brain injury is very complex, including neuronal death, blood–brain barrier injury, white matter axonal injury, cerebral edema, and other causes, which occupies a major position in the risk of neurological deficits and death after ICH [6, 7], and the development of which is closely related to the clinical prognosis of ICH [8, 9]. Current treatments for ICH are limited and focus on the original injury and prevention of rebleeding. These include surgery, cranial pressure reduction, blood pressure reduction, hemostasis, and new nanomedicines. However, these techniques are ineffective in treating secondary brain injury and do not appreciably improve ICH patient prognoses or fatality rates [10]. As a result, current research has focused on reducing subsequent brain injury following ICH.

ICH is caused by an inflammatory response, apoptosis, necrosis, DNA damage, oxidative stress, neuronal toxicity, and reactive oxygen species (ROS), all of which interact to produce secondary brain death [6, 7]. Chen et al. [11, 12] found that Oroxin A and Netrin-1 inhibited ferroptosis through activation of Nrf2/GPX4 and PPARγ/Nrf2/GPX4 pathways, respectively, thereby alleviating subarachnoid hemorrhage and reducing early brain injury in mice. This suggests that ferroptosis may be involved in brain injury after subarachnoid hemorrhage via multiple pathways. Ferroptosis is an important mechanism mediating cell and organ damage, and inhibition of ferroptosis may provide a new approach to alleviate the phenomenon of ischemic cerebral infarction. Ferroptosis is a unique mode of cell death driven by iron-dependent phospholipid peroxidation, characterized by an increase in free ferrous ions and the accumulation of lipid peroxides until fatal cell levels are achieved [13–15]. It involves three essential elements: oxidized lipids, ROS, and lipid peroxidation (LPO) [16, 17]. Ferroptosis is precisely regulated at several levels, including post-translational modifications (PTM) and epigenetic modifications (i.e., regulation of gene expression without altering DNA sequences, including DNA methylation, histone modifications, and non-coding RNAs) [18]. During ICH, a large number of erythrocytes enter the cellular interstitial space of extravascular brain tissue from blood vessels, and the disintegration of erythrocytes releases hemoglobin, which enters neurons and generates substantial LPO [19, 20]. In addition, the increased permeability of the blood–brain barrier leads to infiltration of various Fe3+-rich components of the blood into the brain parenchyma. Fe3+-TF can bind to TfR1 on the surface of brain cell membranes and enter cells through endocytosis, where Fe3+ is reduced to Fe2+ and translocated to the cytoplasm via DMT1. Moreover, Fe2+ triggers the Fenton reaction to form ROS and affects the catalytic activity of lipoxygenase (LOX) [21]. This process is most likely to be an important reason for the irreversible death of neurons caused by secondary injury that occurs after ICH [6, 22]. Therefore, inhibiting ferroptosis is a key strategy for treating secondary brain injury following ICH. Exploring the pathophysiological mechanisms underlying the occurrence of neuronal ferroptosis after ICH, as well as searching for potential means and drugs to inhibit it, are not only current challenges for the medical community but also important issues related to our country’s social and economic development and resolving livelihood issues.

Platelet factor 4 (PF4/CXCL4), a protein derived from platelet alpha granules, is a tetramer of basic polypeptides containing 70 amino acids [23]. PF4 has a number of biological roles, including the suppression of vascular endothelial cell development, modulation of platelet aggregation, and antibacterial and antiviral activities. In addition, PF4 plays a role in physiological processes, such as inflammation, immunological modulation, and angiogenesis [24, 25]. In recent years, researchers have continuously and extensively researched the link between PF4 and inflammation and aging [26–29] and confirmed the protective effect of PF4 on ICH [30]; however, the related mechanisms have not been adequately investigated. In our preliminary experiments, we found that PF4 reduced ICH neuroinflammation. However, it is unclear whether PF4 affects ICH-induced neuronal ferroptosis and its mechanism of action requires further investigation.

In this study, we analyzed the effect of PF4 on neuroinflammation (the cause of secondary brain injury) after ICH by constructing cellular and mice ICH injury models, investigated whether PF4 attenuates ICH injury and inflammatory responses in mice by inhibiting neuronal ferroptosis in the brain, and elucidated the potential regulatory mechanisms by which PF4 affects ICH injury, with the goal of providing theoretical references for future ICH prevention and treatment efforts.

## Materials and methods

### Experimental cell culture and in vitro ICH model creation

Rat adrenal pheochromocytoma PC12 cells were obtained from the Chinese Academy of Sciences Cell Resource Center (Shanghai, China). PC12 cells were grown in RPMI-1640 medium with 10% fetal bovine serum at 37 ^∘^C in a 5% CO_2_ incubator. The medium was replaced every two days. PC12 cells were grown until fusion reached 70%–80%, then treated with 5 µmol/L chloroferritin hemin (Sigma-Aldrich, St. Louis, MO, USA) for 24 h. An in vitro ICH model was created.

PC12 cells were randomly divided into eight groups: control (normal control group), Erastin (ferroptosis induction group, HY-15763, 10 µmol/L, MedChemExpress, Monmouth Junction, NJ, USA), hemin (ICH model group, HY-19424, 5 µmol/L), hemin+Z-VAD (ICH model + Caspase inhibitor treatment group, HY-16658B, 20 µM), hemin+NEC-1 (ICH model + Necrotic apoptosis inhibitor treatment group, HY-15760, 20 µM), hemin+Fer-1 (ICH model + Ferroptosis inhibitor treatment group, HY- 100579, 20 µM), hemin+Rm-PF4 (ICH model+PF4 protein treatment group, HY-P71885, 10 ng), and hemin+Rm-PF4+AMG487 (ICH model+PF4 protein+CXCR3 antagonist treatment group, HY-15319, 1 µM), followed by subsequent experiments.

### Cell Counting Kit-8 (CCK-8) detection

PC12 cells in logarithmic growth phase were seeded in 96-well plates at a density of 1 × 104 cells/well. Culture medium (200 µL) was added to each well and the cells were cultivated for 48 h under various treatment conditions. Then, 20 µL of the CCK-8 reagent solution (HY-K0301, MedChem Express) was added to each well, mixed thoroughly, and incubated for 2 h. The cell proliferation viability in each well was measured using the DR-200Bc enzyme labeling instrument (Diateklab, Jiangsu, China) with the formula: “(OD450 nm value of treatment group/OD450 nm value of control group) × 100%”.

### 5-Ethynyl-2’-deoxyuridine (EdU) detection

The proliferation of PC12 cells was measured using an EdU kit (C10310-1; RiboBio, Guangzhou, China). Cells were seeded in 24-well plates at 2 × 104 cells/well and grown under various conditions for 24 h. The culture medium was discarded. The cells were rinsed in PBS and stained with 10 µM EdU solution for 1 h in the dark. The cells were rinsed in PBS, fixed, and permeabilized with 4% paraformaldehyde and 0.3% Triton X-100. The cells were then incubated with the Click reaction solution for 30 min at room temperature while protected from light, followed by staining the nuclei with DAPI for 10 min, sealing the slices, and observing and photographing them with a fluorescence microscope (Leica, Wetzlar, Germany) to assess the level of cell proliferation (the percentage of EdU-positive cells).

### Calcein/PI detection

Calcein/PI kit (C2015M, RiboBio) was used to determine the live/dead cell state. Aspirate off the adherent cell culture medium, wash the cells with PBS once, add the appropriate volume of Calcein AM/PI assay working solution, and incubate at 37 ^∘^C in the dark for 30 min. After incubation, the staining results were examined using a fluorescence microscope (Calcein AM is green fluorescence, Ex/Em ═ 494/517 nm; PI is red fluorescence, Ex/Em ═ 535/617 nm).

### Immunofluorescence staining

PC12 cells were injected into 6-well plates and immunofluorescence was used to identify the cells once approximately 80% cell attachment was observed. PC12 cells were treated with hemin and Ferrostatin-1 (Fer-1) before fixation with 4% paraformaldehyde, permeabilization with 0.1% Triton X-100, and closure with 2% BSA. PC12 cells were treated with rabbit anti-GPX4 (1:500, ab125066, Abcam, Waltham, MA, USA) at 4 ^∘^C overnight. The cells were rinsed with PBS and treated with Alexa Fluor^®^ 488 labeled sheep anti-rabbit IgG (1:200, ab150077) for 1 h at 37 ^∘^C. The nuclei were stained with DAPI for 5 min, and the number of GPX4-positive cells was counted using a fluorescence inverted microscope after washing with PBS and applying an anti-fluorescence quencher dropwise.

### In vivo experiment

Chengdu Dossy Experimental Animals Co. supplied healthy male SPF-grade C57BL/6 J mice at eight weeks of age. All mice were housed under a 12-h cycle of light and darkness. Room temperature was maintained at 23 ^∘^C–25 ^∘^C and humidity at 50%–60%, with free access to food and drink. An ICH C57BL/6 mouse model was created using collagenase injection [31]. Mice were anesthetized with 3% sodium pentobarbital intraperitoneally and fixed to a stereotaxic device. The heads of the mice were shaved and sanitized and an incision measuring approximately 5 mm in length was made along the midline of the scalp to fully expose the anterior fontanelle. An electric drill was used to drill a round hole of 1 mm diameter at 0.6 mm anterior to the fontanelle and 3 mm next to the midline to the depth of the dural surface without injuring the brain tissue. Collagenase IV (12.5 U/mL, HY-E70005D, MedChem Express) was added in 2 µL using a microsyringe. A microsyringe was mounted on top of the stereotaxic apparatus. Collagenase IV was injected by vertically inserting the needle tip along the drilled membrane to a depth of approximately 6 mm. The solution was slowly injected at a rate of 10 µL/min and then left for 10 min. The needle was slowly withdrawn, the cranial foramen was closed with bone wax, the wounds were sanitized and sutured, and the body temperature of the rats was maintained at 37 ^∘^C using a thermostatic blanket until they awoke. The mice were divided into three groups: ICH, ICH+Rm-PF4, ICH+Rm--PF4+Fer-1, and ICH+Rm--PF4+AMG487. Each group consisted of six animals. Model mice in the ICH+Rm--PF4 group were injected daily with 100 µL of 5 µg/mL of PF4, model mice in the ICH+Rm--PF4+Fer-1 group were injected daily with 100 µL of 5 µg/mL of PF4 and 2 mg/kg of the mice weight of Fer-1 (dissolved in DMSO), and model mice in the ICH+Rm--PF4+AMG487 group were injected daily with 100 µL of 5 µg/mL of PF4 and 5 mg/kg mice weight of Fer-1, injected continuously for 1, 3, and 7 days, respectively. After completing the neurological function score at the specified time point, the mice were anesthetized, decapitated, dissected, removed from the cranium to extract brain tissues, and stored at −80 ^∘^C.

### Neurological function assessment in mice

The Cylinder and Corner tests were used to evaluate brain neural function in mice under various treatment conditions. According to previous testing methods [21, 32], the mice were placed in a transparent Plexiglas cylinder (the shape of the cylinder could stimulate the rats to explore the wall of the cylinder vertically with their forelimbs), and the number of times the left and right forelimbs, as well as both limbs, touched the wall of the cylinder when 20 mice stood on their hind limbs was recorded. The cylinder test was expressed as (right limb-left limb)/(right limb+left limb+both limbs). The mice were placed in a 30∘ corner and encouraged to turn left or right. Their turn choices were recorded 20 times, and the Corner test was expressed as (number of left turns/number of all turns × 100%). Larger test values indicate more severe left hemiparesis in mice, which translates to more severe neurological impairment.

### HE staining

Mice brain tissues were dehydrated in ethanol, made transparent in xylene, and wax-dipped before being cut into 5 µm paraffin sections with a slicer. Paraffin slices were cooked in a baking machine at 65 ^∘^C for 2 h, deparaffinized with xylene, and rehydrated using an alcohol gradient. Sections were stained with hematoxylin (C0107, Beyotime, Shanghai, China) for 15 min, differentiated with 1% acidic alcohol (containing 70% hydrochloric acid) for 30 s, washed with running water, and submerged in 0.5% eosin (G1100, Solarbio, Beijing, China) for 3 min. Following staining, the sections were dehydrated using an alcohol gradient, cleared with xylene, and sealed with neutral gum (G8590; Solarbio). Finally, the tissue morphology of the slices was examined under a microscope.

### TUNEL fluorescent staining

Apoptosis in brain tissues was assessed using the TUNEL kit (C1091, Beyotime) according to the manufacturer’s instructions. Tissue slices were deparaffinized with xylene and hydrated with gradient ethanol. Then, 20 µg/mL of DNase-free proteinase K solution was added and incubated at room temperature for 30 min to promote reaction reagents to enter the nucleus. After washing in PBS, tissue sections were submerged in 3% H_2_O_2_ for 15 min to deactivate endogenous peroxidase in the sections. Sections were washed with PBS, stained with 50 µL of TUNEL staining solution at 37 ^∘^C for 60 min, rinsed with PBS, stained with DAPI staining solution (C1005, Beyotime) at room temperature for 5 min, and protected from light. Sections were rinsed with PBS, blocked with an Anti-fluorescence Burst Sealer, and the percentage of TUNEL-positive cells was determined through microscopic inspection.

### Nissl staining

Paraffin slices of mice brain tissues were stained with 1% toluidine blue (G3668, Solarbio) at 60 ^∘^C for 30 min. Ethanol gradient dehydration was carried out, and xylene was used for transparency before being sealed with neutral gum. Finally, loss of Nissl bodies in the brain tissue was observed and photographed using a light microscope.

### IHC

Brain tissue sections were dehydrated with ethanol, antigenically repaired with citrate (C1032, Solarbio), and blocked with avidin/biotin blocking buffer (C-0005, HaoRan Biotech, Shanghai, China) at room temperature before adding the primary antibodies Iba-1 (1:2000, ab178846), CD16 (1:500, ab198507), and Arg1 (1:5000, ab315110), and incubated at 4 ^∘^C overnight. After washing, the appropriate secondary antibody (1:500, ab150077, Abcam) was applied to the tissue sections and incubated for 1 h at room temperature. The sections were restained with a streptavidin–horseradish peroxidase combination and hematoxylin (C0107, Beyotime). Slices were dehydrated using an ethanol concentration gradient, treated with xylene, and sealed with neutral gum. Sections were observed under a microscope and photographed.

### ROS levels detection

Cellular ROS levels were measured using an S0033S kit (Beyotime). The ROS staining solution (10 µM DCFH-DA) was prepared according to the manufacturer’s instructions. The solution was mixed well with each group of PC12 cells (1 × 106), incubated in a 6-well plate at 37 ^∘^C for 20 min, protected from light, centrifuged to remove the supernatant, washed, and resuspended in PBS. The cells were filtered to produce a single-cell solution for flow cytometry. The cells were collected at 1 × 10^5^ cells as the termination condition, and the fluorescence intensity was recorded at an excitation wavelength of 488 nm and an emission wavelength of 525 nm within 30 min. Changes in ROS intensity were detected using FlowJo software. Mice brain tissues were frozen and sliced into 10 µm sections within 1 h. ROS levels were measured using an HR9069 kit (Baiao Leibo, Beijing, China). The staining and washing working solutions were prepared by diluting the DHE probe and washing solution, respectively, with pure water. Apply 200 µL of washing solution to the entire slice and incubate at room temperature for 3–5 min. The washing solution was pipetted, 100 µL of the staining solution was applied to the slice, and incubated at 37 ^∘^C for 20 min in the dark. The staining solution was pipetted and washed with PBS before sealing the segment with glycerol, visualizing under a fluorescence microscope, and analyzing using FlowJo software.

### Testing with detection kits

The following kits were used for detection: malondialdehyde (MDA) (BC0025, Solarbio), GSH (BC1175, Solarbio), myeloperoxidase (MPO) (BC5710, Solarbio), and Fe2+ (BC5415, Solarbio). PC12 cells were washed with PBS and the number of cells was combined with the volume of the extraction solution at a 500:1 ratio. The cells were shattered using ultrasonic waves in an ice bath, and the supernatant was collected after centrifugation at 4 ^∘^C for 10 min. Brain tissue was weighed and homogenized in PBS on ice. The supernatant was collected by centrifugation at 4 ^∘^C for 10 min. The corresponding reagents were prepared as directed and mixed with PC12 cells/tissue supernatants. The absorbance of each group of samples at different wavelengths was measured using an enzyme labeling apparatus, and the relative levels of MDA, GSH, MPO, and Fe2+ in PC12 cells and mice brain tissues were calculated.

### ELISA detection

PC12 cells and mice brain tissues were tested with ELISA (enzyme-linked immunosorbent assay). IL-6, TNF-α, and IL-1β concentrations were measured using Beyotime’s PI326, PT512, and PI301 kits, following their instructions. The plate was incubated with 100 µL of sample analysis buffer at 37 ^∘^C for 2 h. Next, 100 µL biotinylated antibody was added to the plate and incubated at 37 ^∘^C for 1.5 h. Next, 100 µL of affinity hormone–horseradish peroxidase marker was added and incubated at 37 ^∘^C for 20 min. Next, 100 µL of TMB chromogenic solution was added and incubated for 20 min in the dark. Finally, the plate was supplemented with 50 µL of termination solution to stop the reaction. The absorbance of the samples was measured at 450 nm using an enzyme marker and the content was estimated.

### Perls staining

The DAB-enhanced Perls staining kit (G1428, Solarbio) was used to detect iron accumulation. Brain tissue sections were washed with PBS and H_2_O_2_ before being incubated for 1 h in newly prepared Perls solution (solution A: solution B ═ 1:1), followed by three PBS washes. The cells were then incubated with DAB for 3 min. The exact time depended on the depth of staining. Free iron ions in the brain tissue are blue, while normal tissue is red. Following staining, the slices were sealed with neutral glue and examined using a fluorescent inverted microscope to determine the deposition of comparable iron ions, which were counted using ImageJ software.

### Western blot

Total protein in mice brain tissues and PC12 cells was extracted from cell lysate, the concentration of each group of proteins was determined using a BCA protein detection kit, separated by SDS-PAGE after quantitative denaturation, and the proteins were quickly transferred to a PVDF membrane and placed in TBST solution containing 5% skimmed milk at room temperature for 2 h. Rabbit anti-GPX4 (1:1000, ab125066), FTH1 (1:1000, ab75972), ACSL4 (1:10000, ab155282), COX-2 (1: 1000, ab179800), CXCR3 (1:1000, ab288437), AKT1 (1: 1000, ab314110), p-AKT1 (1: 5000, ab81283), EZH2 (1: 1000, ab307646), p-EZH2 (1: 1000, ab300567), H3K27me3 (1: 1000, ab192985), SLC7A11 (1: 1000, ab307601), and GAPDH (1:10000, ab9485) were each added at 4 ^∘^C overnight. After three TBST washes, the membrane was incubated with IgG (1:2000, ab205718) for 2 h at room temperature. Finally, DAB colorant (DA1016, Solarbio) was used to develop the color, and the relative expression levels of the proteins in each group were quantified using a gel imager (5200, Tanon, Shanghai, China) to record protein grayscale and snap images, with GAPDH serving as a control.

**Figure 1.**
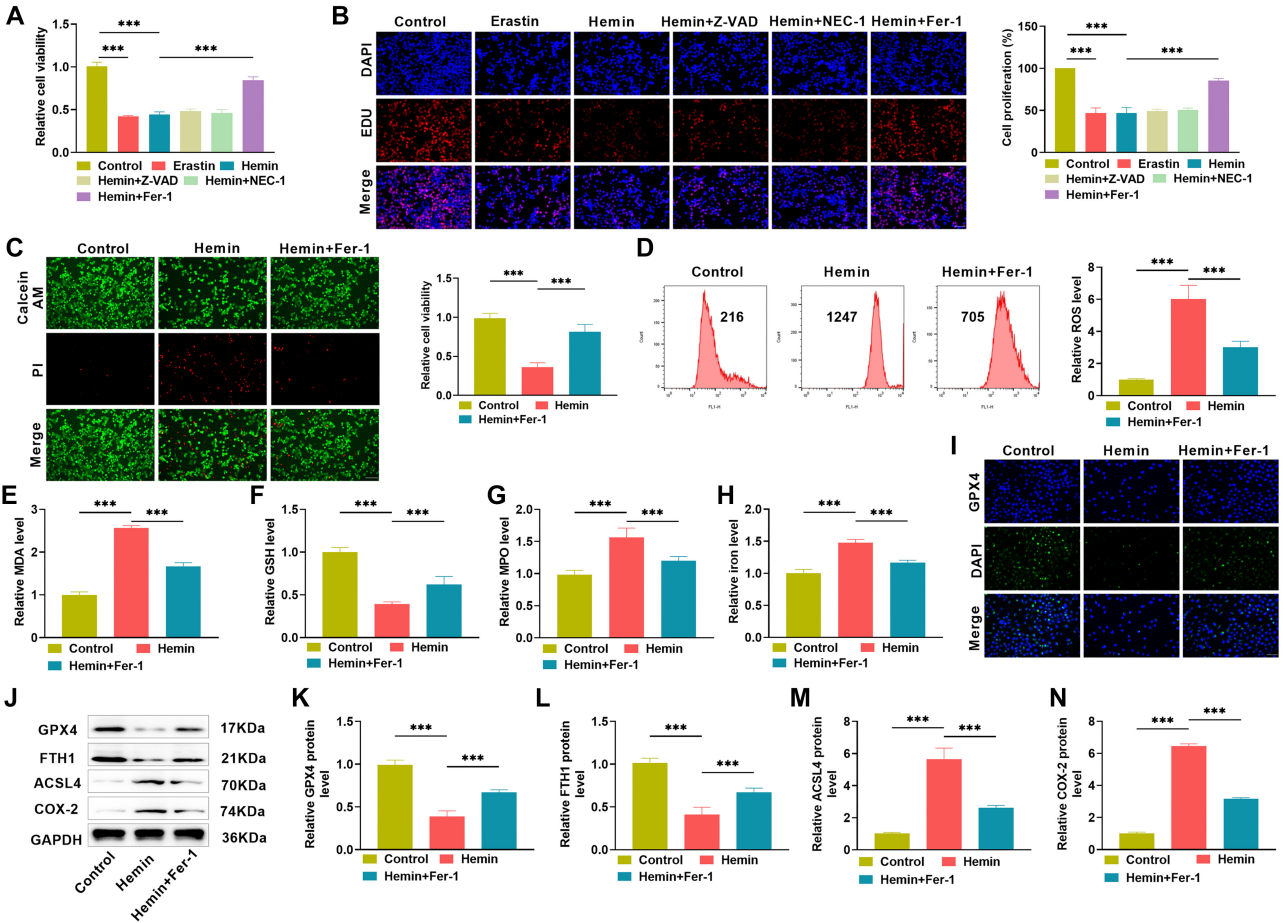
**Hemin induces ferroptosis in PC12 cells.** (A and B) PC12 cells were treated with erastin, hemin, and various inhibitors, and changes in cell proliferation viability were measured using CCK-8 and EdU assays to speculate on Hemin-induced effects; (C) To examine apoptosis, the red/green staining effect (PI/Calcein AM) of PC12 cells treated with varied circumstances was detected using the calcein/PI kit and fluorescence microscopy; (D) Use flow cytometry to detect changes in ROS levels in PC12 cells following treatment with different conditions; (E–H) PC12 cells were subjected to various treatments, and the levels of lipid peroxides MDA and MPO, antioxidants GSH, and iron ions were measured using the relevant kits; (I) Immunofluorescence detection of GPX4 expression in PC12 cells from various treatment groups; (J–N) Western blot to measure changes in the levels of ferroptosis-related proteins GPX4, FTH1, ACSL4, and COX-2. CCK-8: Cell counting kit-8; EdU: 5-Ethynyl-2’-deoxyuridine; ROS: Reactive oxygen species; MPO: Myeloperoxidase; Fer-1: Ferrostatin-1; MDA: Malondialdehyde.

### Ethical statement

This study was approved by Hebei University of Chinese Medicine (DWLL202402091).

### Statistical analysis

The data were analyzed using SPSS 26.0 software (SPSS Inc., Chicago, IL, USA) and plotted with GraphPad Prism 9.0 software (GraphPad Inc., La Jolla, CA, USA). The measurements were represented as ±s. Independent *t*-tests were employed between two groups, whereas one-way ANOVA was used between multiple groups, followed by Tukey’s post hoc test. Data were normally distributed. A *P* value < 0.05 indicates a significant difference.

**Figure 2.**
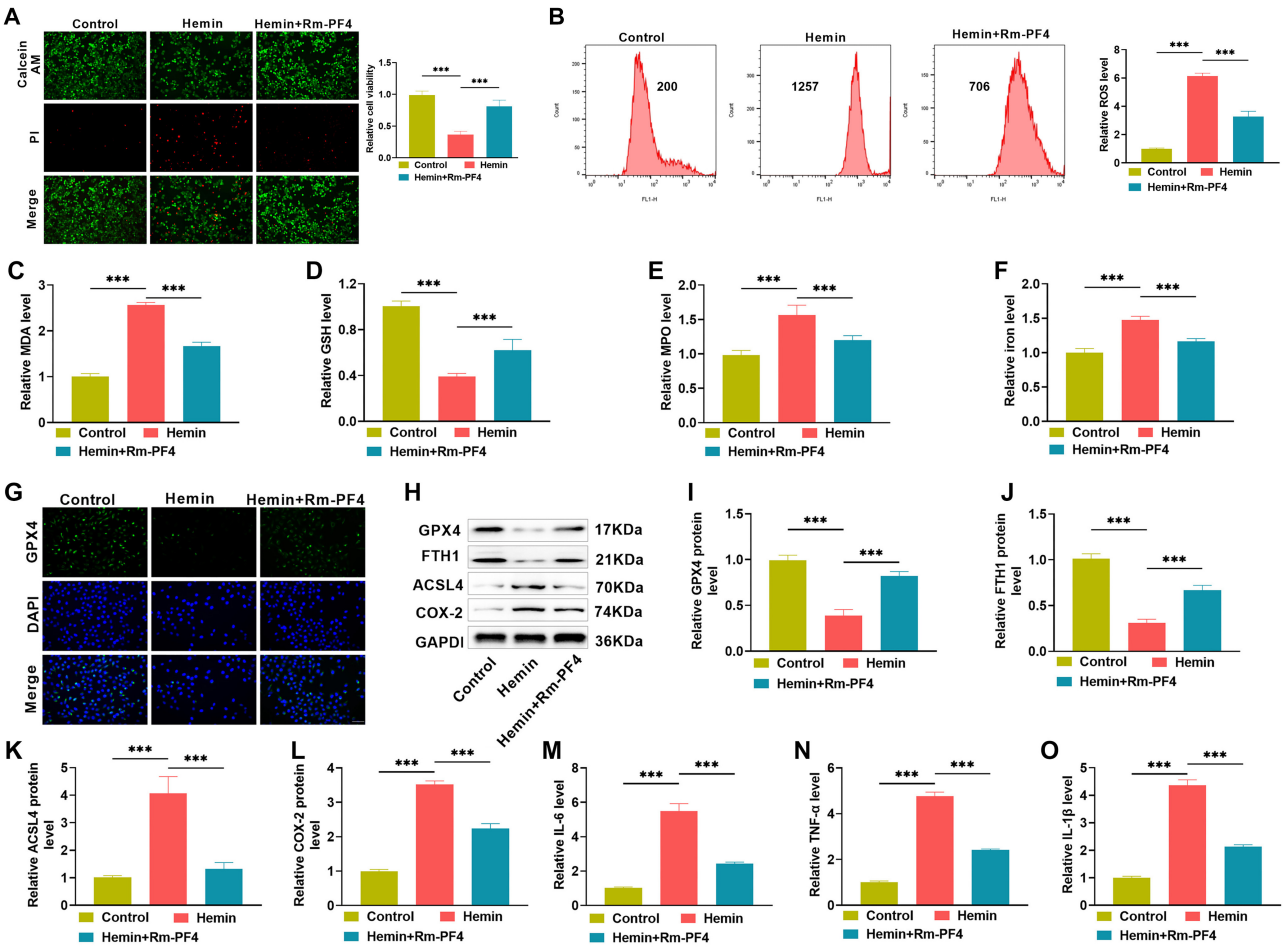
**The addition of exogenous PF4 recombinant protein reduces ferroptosis-mediated Hemin-induced PC12 cell damage and inflammation.** (A) The calcein/PI kit was used to identify cell death after PF4 treatment in a Hemin-induced; (B) A flow cytometry test to determine the influence of PF4 on ROS levels in ICH cells; (C–F) Kits were used to measure the effects of PF4 on the levels of MDA, GSH, MPO, and iron ions in ICH cells; (G) Immunofluorescence to assess changes in the number of GPX4-positive cells in ICH cells treated with PF4; (H–L) Western blot to see how PF4 affects the levels of GPX4, FTH1, ACSL4, and COX-2 in ICH cells; (M–O) ELISA to measure the levels of inflammatory factors IL-6, TNF-α, and IL-1β in ICH cells treated with PF4. ROS: Reactive oxygen species; PF4: Platelet factor 4; Rm-PF4: Recombinant PF4; ICH: Intracerebral hemorrhage; MPO: Myeloperoxidase; MDA: Malondialdehyde.

## Results

### Hemin induces ferroptosis in PC12 cells

PC12 cells are rat adrenal medullary chromaffinoma differentiated cell lines with general characteristics of neural cells and are widely used in neuroscience-related in vitro studies. In this study, we used hemin to induce PC12 cells to act as an in vitro ICH model. They were administered an apoptosis inhibitor (Z-VAD), a cell necrosis inhibitor (NEC-1), and a cellular ferroptosis inhibitor (Fer-1) and cultivated for 24 h. Figure 1A depicts the relative vitality of PC12 cells. Compared to the control group, the cell viability of both the erastin ferroptosis-induced group and the ICH model group was significantly reduced (*P* < 0.05), and the cell viability of the ICH model cells did not change significantly after treatment with Z-VAD or NEC-1, whereas it was significantly upregulated after treatment with Fer-1 (*P* < 0.05), indicating hemin-induced ferroptosis in PC12 cells. The EdU assay exhibited the same trend (Figure 1B), demonstrating that hemin induces iron mortality in PC12 cells. Iron deposition, GSH antioxidant failure, GPX4 depletion, and lipid peroxide buildup are biochemical characteristics of ferroptosis, whereas accumulation of cytotoxic chemicals results in protein breakdown, lipid degradation, and cell death. We examined cell viability using calcein/PI fluorescence staining, and the results, as shown in Figure 1C, revealed that hemin-induced a decrease in the number of viable ICH cells and an increase in the number of dead cells, which was reversed following Fer-1 treatment. Later, ROS intensity and oxidative stress indicators in the cells were detected by flow cytometry and kits, respectively, and it was found that hemin significantly increased ROS intensity (Figure 1D), MDA (Figure 1E), MPO levels (Figure 1G), and promoted iron deposition (Figure 1H), while significantly decreasing the antioxidant glutathione GSH (Figure 1F), and the key ferroptosis protein GPX4 was significantly reduced after hemin induction (Figure 1I) (*P* < 0.05). Following quantitative investigation by western blotting, hemin triggered PC12 cells with lower levels of GPX4 and FTH1 and elevated levels of ACSL4 and COX-2, suggesting ferroptosis (Figure 1J–1N). Taken together, these experimental data showed that hemin caused iron mortality in PC12 cells.

### PF4 recombinant protein inhibits hemin-induced PC12 cell damage and inflammatory responses

To evaluate the effect of the PF4 recombinant protein Rm-PF4 on hemin induction, we treated ICH model cells with 10 ng Rm-PF4. The experimental results showed that PC12 cell mortality and ROS levels were much lower in the hemin+Rm-PF4 group than in the hemin alone induction group (Figure 2A and 2B), and the levels of lipid peroxides MDA and MPO, as well as iron ions, were dramatically reduced, whereas the antioxidant GSH increased (Figure 2C–2F), while the levels of ferroptosis-related proteins GPX4 and FTH1 were increased, while ACSL4 and COX-2 were decreased (Figure 2G–2L). Rm-PF4 therapy significantly reduced the levels of the cellular inflammatory markers IL-6, TNF-α, and IL-1β, as evaluated by ELISA (Figure 2M–2O), indicating that it reduced ferroptosis-mediated hemin-induced PC12 cell damage and inflammation.

### PF4 regulates the CXCR3/AKT1/SLC7A11 signaling pathway

**Figure 3.**
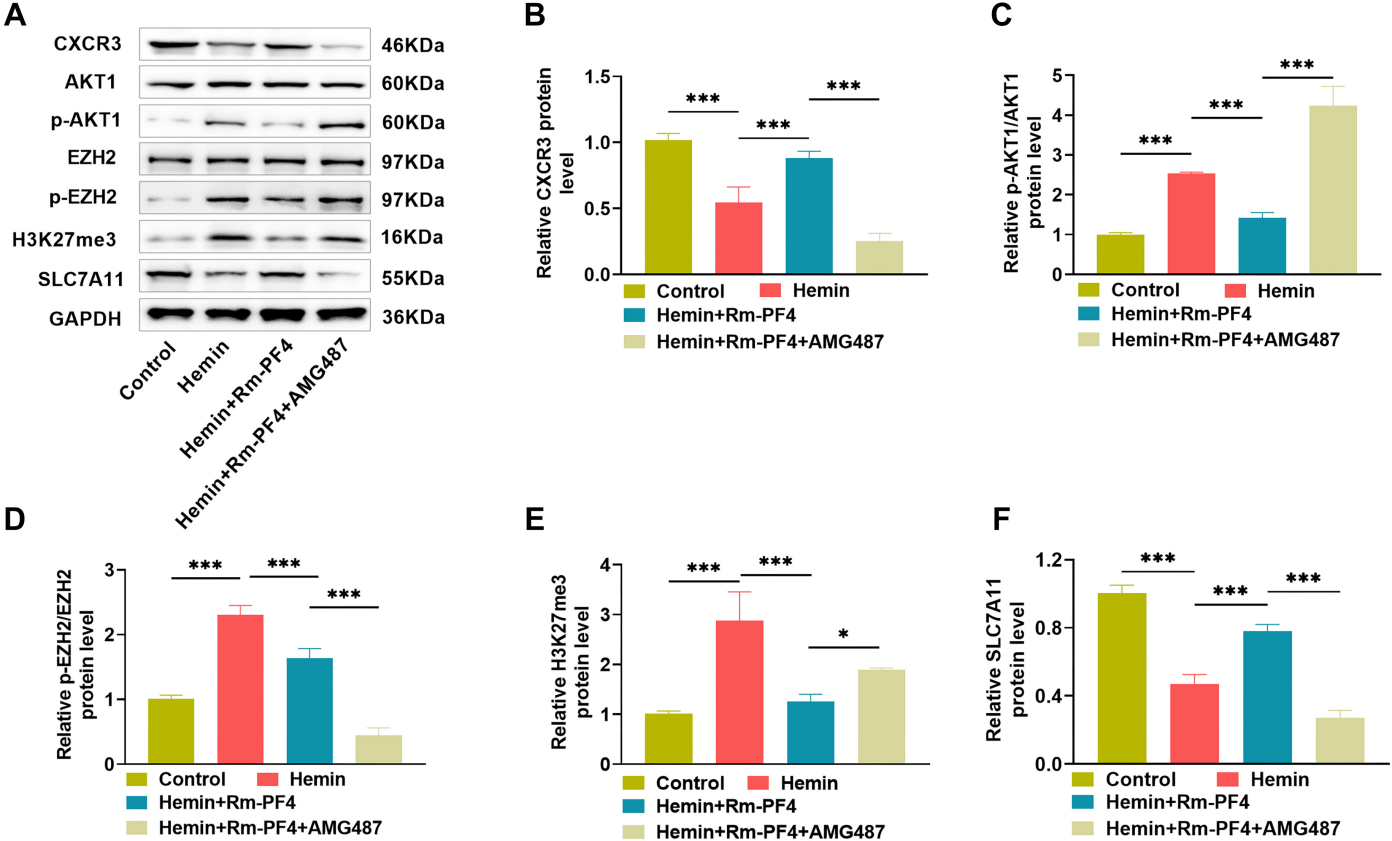
**PF4 regulates the CXCR3/AKT1/SLC7A11 signaling pathway.** (A–F) Western blot analysis of protein expression in PC12 cells under various treatment conditions. PF4: Platelet factor 4; Rm-PF4: Recombinant PF4.

PF4/CXCL4 is known to inhibit the inflammatory response by binding to the receptor CXCR3 [33] and can also inhibit the activation of EZH2 by hindering the phosphorylation of AKT1 through binding to CXCR3, while EZH2 can inhibit the expression of SLC7A11 through histone H3K27me3 modification [34–36]. Based on the present study, we hypothesized that PF4’s mechanism of action in reducing hemin-induced PC12 cell damage is linked to the CXCR3/AKT1/SLC7A11 signaling pathway. To test this hypothesis, we treated ICH model cells with both Rm-PF4 and CXCR3 antagonists before performing the rescue studies. Western blot analysis revealed that hemin alone induced down-regulation of CXCR3 and SLC7A11 protein levels, increased phosphorylation levels of AKT1 and EZH2, and upregulation of histone H3K27me3; however, Rm-PF4 treatment significantly attenuated the above conditions and effectively alleviated the inducing effect of hemin; when CXCR3 antagonist AMG487 was added concurrently, the effect of Rm-PF4 was significantly reversed (*P* < 0.05, Figure 3A–3F). The experimental results revealed that PF4 mostly functions via the CXCR3/AKT1/SLC7A11 signaling pathway, which could be an essential mechanism for its suppression of ferroptosis and reduction of PC12 cell damage and the inflammatory response.

### Inhibiting the CXCR3/AKT1/SLC7A11 signaling pathway partially reverses PF4’s attenuating effect on PC12 cell damage and inflammatory response

**Figure 4.**
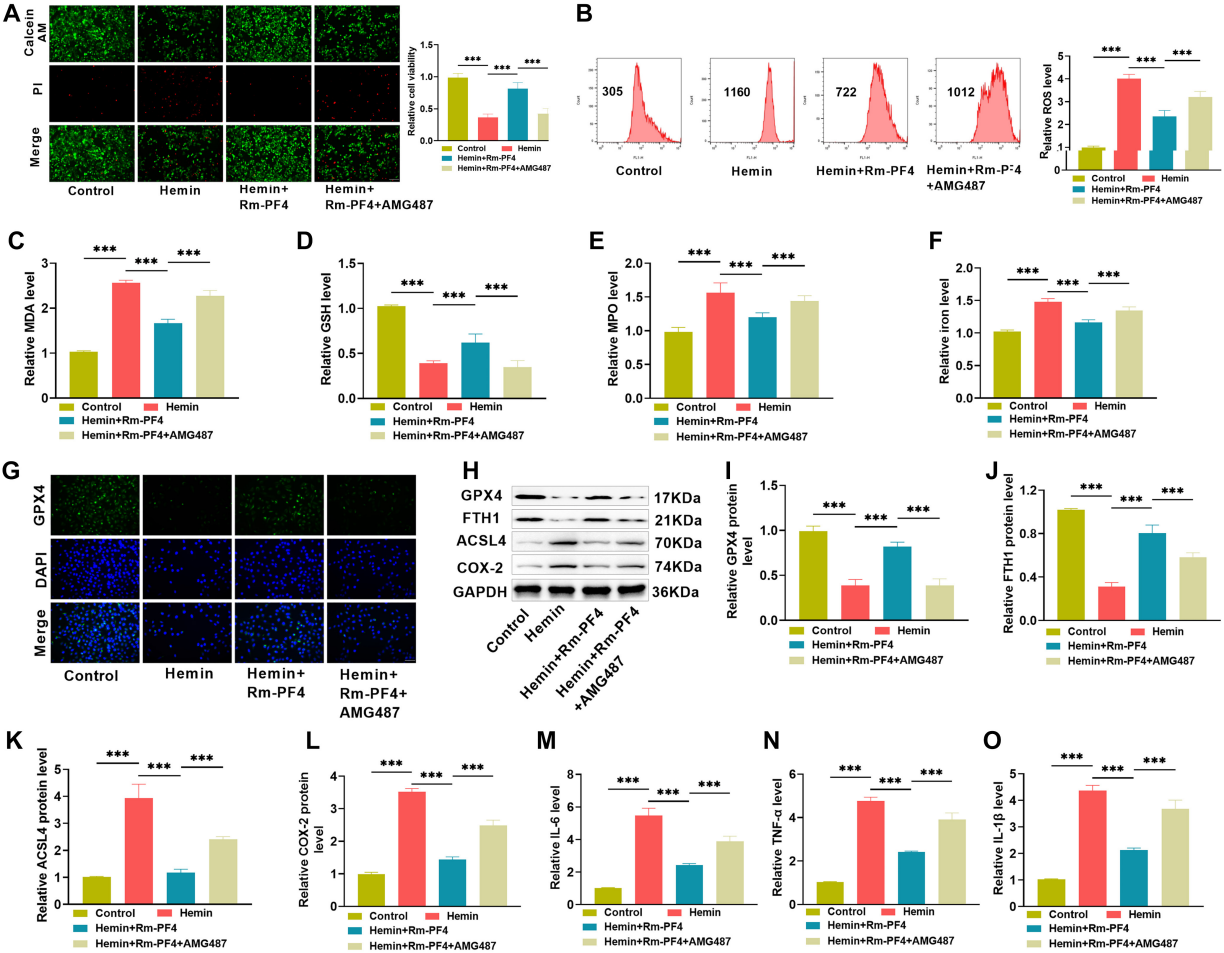
**Inhibition of the CXCR3/AKT1/SLC7A11 signaling pathway partially reverses PF4’s mitigation of ferroptosis-mediated hemin-induced PC12 cell damage and inflammation.** (A) PC12 cells were treated with CXCR3 antagonist AMG487 to validate the PF4 regulation mechanism using the calcein/PI cell viability test; (B) Flow cytometric experiment was used to determine the effect of the antagonist AMG487 on ROS levels in PC12 cells; (C–F) Kits for measuring the effect of the antagonist AMG487 on MDA, GSH, MPO, and iron ion levels in PC12 cells; (G) Immunofluorescence used to identify the effect of the antagonist AMG487 on GPX4 expression in PC12 cells; (H–L) Western blot analysis of the antagonist AMG487’s effect on ferroptosis-related proteins GPX4, FTH1, ACSL4, and COX2; (M–O) ELISA for examing the effect of antagonist AMG487 on inflammatory factor levels in PC12 cells. ROS: Reactive oxygen species; PF4: Platelet factor 4; Rm-PF4: Recombinant PF4; MPO: Myeloperoxidase; MDA: Malondialdehyde.

We used AMG487 to suppress the CXCR3/AKT1/SLC7A11 signaling pathway and investigated the effect of PF4 on PC12 cell survival, oxidative stress, ferroptosis, and inflammation. Figure 4A–4O displays the results: inhibiting the CXCR3/AKT1/SLC7A11 pathway in PC12 cells led to decreased cell viability, increased oxidative stress (elevated levels of ROS, MDA, MPO, and ferric ions, and decreased levels of GSH), increased ferroptosis (downregulation of GPX4 and FTH1 levels, and upregulation of ACSL4 and COX-2 levels), and increased inflammatory response (elevated IL-6, TNF-α, and IL-1β levels). These data indicate that PF4 inhibits PC12 cell damage and inflammatory responses by activating the CXCR3/AKT1/SLC7A11 signaling pathway.

### Rm-PF4 mediates ferroptosis to reduce hemorrhagic brain damage in mice

**Figure 5.**
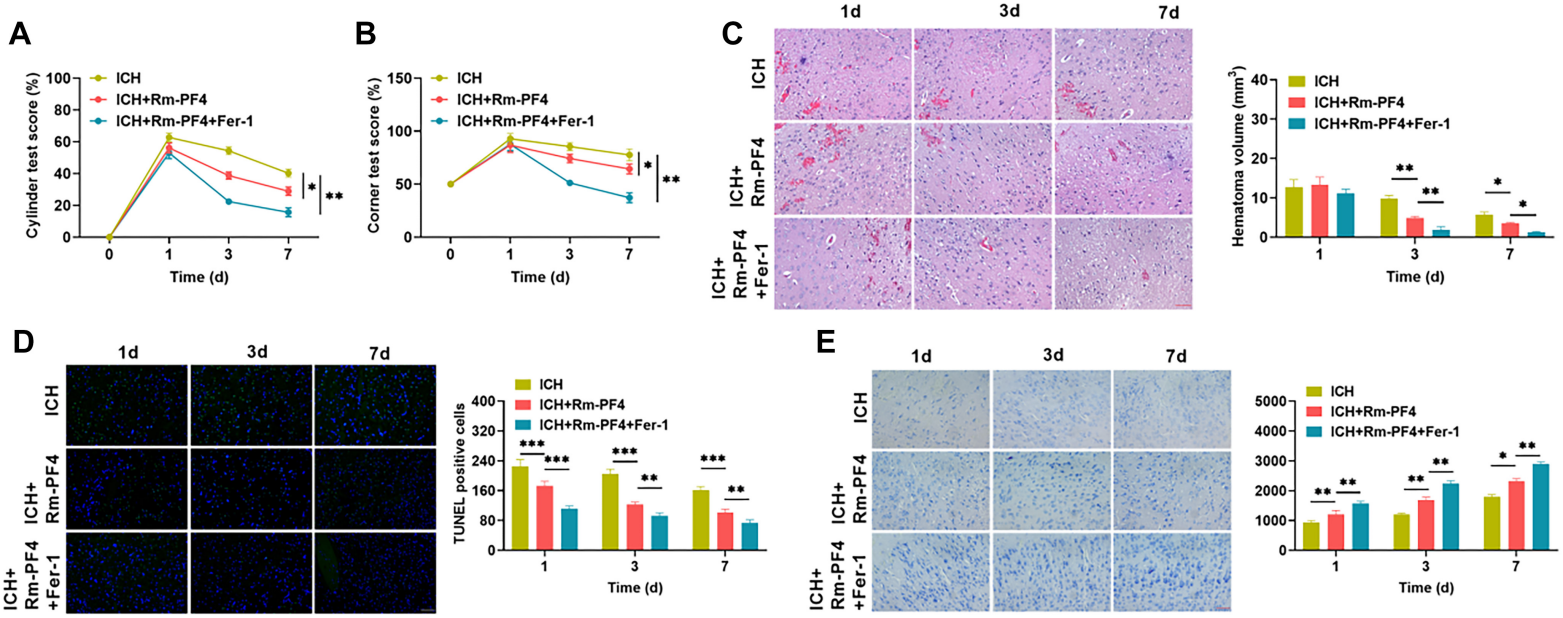
**Rm-PF4 mediates ferroptosis to attenuate ICH injury in mice.** (A and B) ICH mice model was created by injecting collagenase and then administered PF4 and Fer-1. The mice were necropsied on days 1, 3, and 7, respectively. Neurological alterations were assessed using the Cylinder and Corner tests. (C) HE staining was utilized to assess the pathological damage to mice brain tissues under various treatment settings. (D) TUNEL fluorescence labeling to detect apoptosis in tissue cells under various treatment circumstances. (E) Nissl-staining to determine changes in the number of neurons in tissues under various treatment circumstances. PF4: Platelet factor 4; Rm-PF4: Recombinant PF4; ICH: Intracerebral hemorrhage; Fer-1: Ferrostatin-1.

We successfully created an ICH animal model using collagenase injection to explore the effects of Rm-PF4 on ICH in vivo. Following successful modeling, the Cylinder test and Corner test scores were utilized to measure the degree of neurological function damage in each group of mice, after which the mice were sacrificed and brain tissues were collected to prepare tissue slices. HE staining was used to investigate histological changes around the hematoma, TUNEL staining to observe tissue apoptosis, and Nissl staining to detect neuronal injury. As demonstrated in Figure 5A and 5B, the scores of mice in each group followed the same trend, with the highest scores at 1 h of modeling and then a progressive reduction. On the first, third, and seventh days of mice rearing, both Cylinder test and Corner test scores showed ICH+Rm--PF4+Fer-1<ICH+Rm--PF4<ICH, i.e., the number of times the left limb touched the wall of the cylinder, as well as the number of left turns were sequentially reduced, and the neurological impairment of the mice was aggravated, which corresponded to the histopathological damage of HE staining (Figure 5C). TUNEL labeling was used to detect apoptosis in the brain tissue near the hematoma. Fer-1 injection increased the effect of Rm-PF4 while inhibiting the increase in TUNEL-positive cells (Figure 5D). Nissl staining revealed a decrease in the number of neurons in the ICH group, that is, in the tissue surrounding the hematoma after ICH, the nuclei of the cells were condensed, and Nissl granules were not obvious. Following Rm-PF4 injection, there was occasional nuclear condensation of cells, along with an increase in the number of neurons and Nissl granules. The number of neurons increased following Fer-1 inhibition of ferroptosis when compared to the ICH+Rm--PF4 group, the neuronal cell structure remained intact, the nuclei were clearly and abundantly marked, and the cytoplasmic Nissl granules increased (light blue in color) (Figure 5E). Rm-PF4 reduces hemorrhagic brain damage in mice by mediating ferroptosis.

### Rm-PF4 mediates ferroptosis to enhance microglia activation and reduce pro-inflammatory factor release following ICH

**Figure 6.**
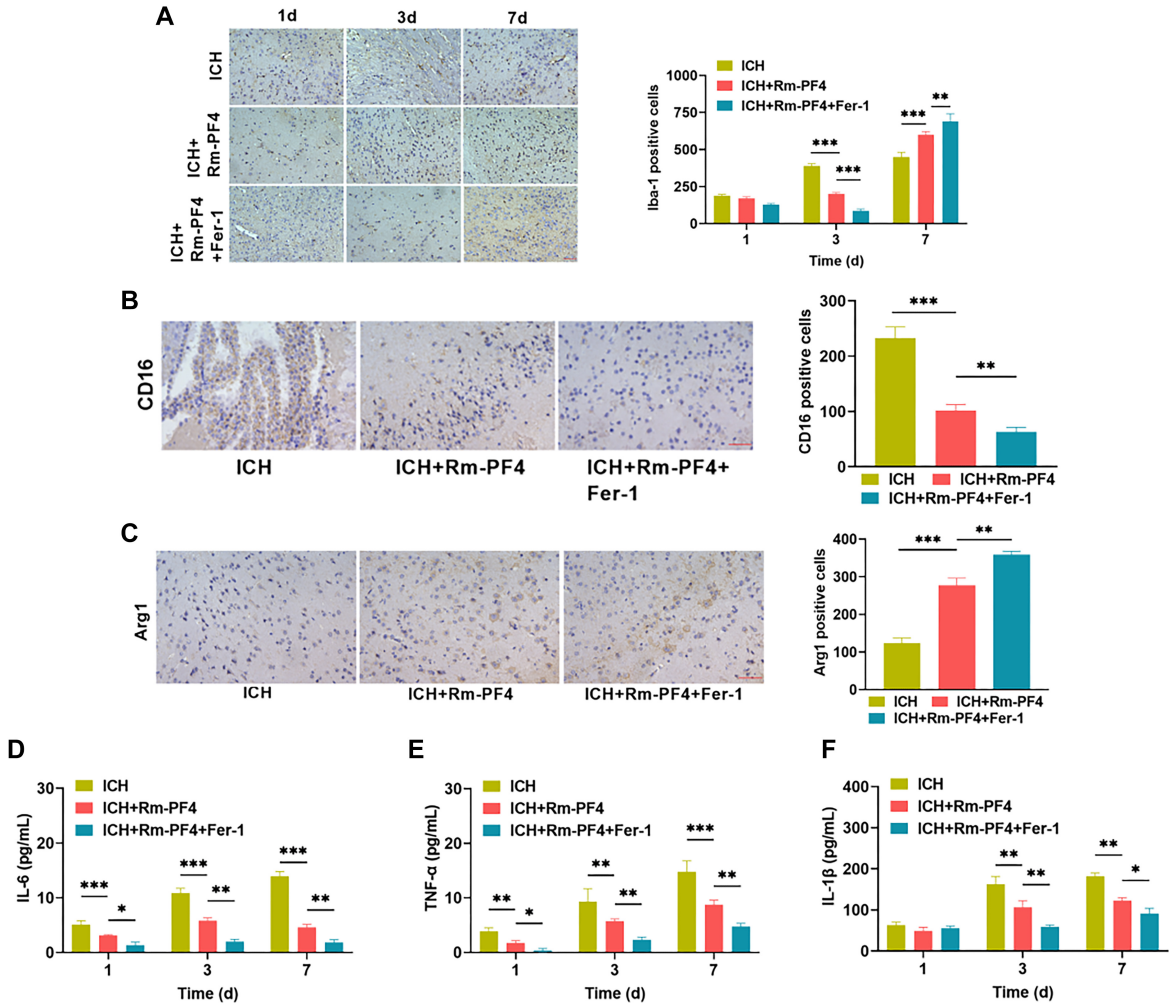
**Rm-PF4 mediates ferroptosis to increase microglia activation and decrease pro-inflammatory cytokine production following ICH.** (A) IHC detected the expression of the microglial cell marker Iba-1 during brain tissue bleeding under each treatment condition; (B and C) On the third and seventh days, IHC revealed the expression of the pro-inflammatory marker CD16 and the anti-inflammatory marker Arg1; (D–F) ELISA for detecting the IL-6, TNF-α, and IL-1β levels in brain tissues. Rm-PF4: Recombinant PF4; ICH: Intracerebral hemorrhage.

Brain injury due to ICH is caused by neuroinflammation, which involves microglial activation and neutrophil infiltration. This leads to the release of pro-inflammatory cytokines, such as IL-6, TNF-α, and IL-1β, as well as MPO and other harmful substances [37, 38]. After ICH, microglia undergo a series of morphological and functional changes from the M0 state and eventually polarize into the M1-type and M2-type microglia, with M1 microglia playing a role in promoting the development of inflammation and M2 microglia playing a role in suppressing inflammation [39]. We determined the polarization status of microglia by analyzing the interference of Rm-PF4 with microglial activation and inflammatory factor release in ICH mice. Immunohistochemical analysis revealed that the microglial marker Iba1 accumulated on day one and increased from day three to day seven [40]. Iba1 levels were considerably lower and higher on days three and seven after Rm-PF4 injection, respectively, compared to the ICH group (*P* < 0.05), which is consistent with previously reported results [41]. Furthermore, injection of Fer-1 improved the effect of Rm-PF4 (Figure 6A), implying that Rm-PF4 could inhibit ferroptosis to boost microglial activation. On days three and seven, there was positive expression of the pro-inflammatory marker CD16 and the anti-inflammatory marker Arg1, as shown in Figure 6B and 6C, Rm-PF4 significantly reduced the expression of the pro-inflammatory marker CD16 and increased the expression of the anti-inflammatory marker Arg1 in ICH tissues (*P* < 0.05), which was further facilitated by the inhibition of ferroptosis by Fer-1 injection, implying that microglia are predominantly polarized to the M2 phenotype, and ferroptosis-induced microglial inhibition suppressed neuroinflammatory responses. ELISA was used to identify changes in the levels of IL-6, TNF-α, and IL-1β in brain tissues. The ICH+Rm--PF4 group showed considerably lower levels of pro-inflammatory markers compared to the ICH group (*P* < 0.05). This downregulation of IL-6, TNF-α, and IL-1β persisted after Fer-1 blocked ferroptosis (Figure 6D–6F). The experimental results indicated that Rm-PF4 inhibits microglial M2 activation during ICH by inducing ferroptosis, which restricts the release of pro-inflammatory molecules and reduces neuroinflammation.

### Rm-PF4 affects the CXCR3/AKT1/SLC7A11 signaling pathway to reduce ferroptosis after ICH

**Figure 7.**
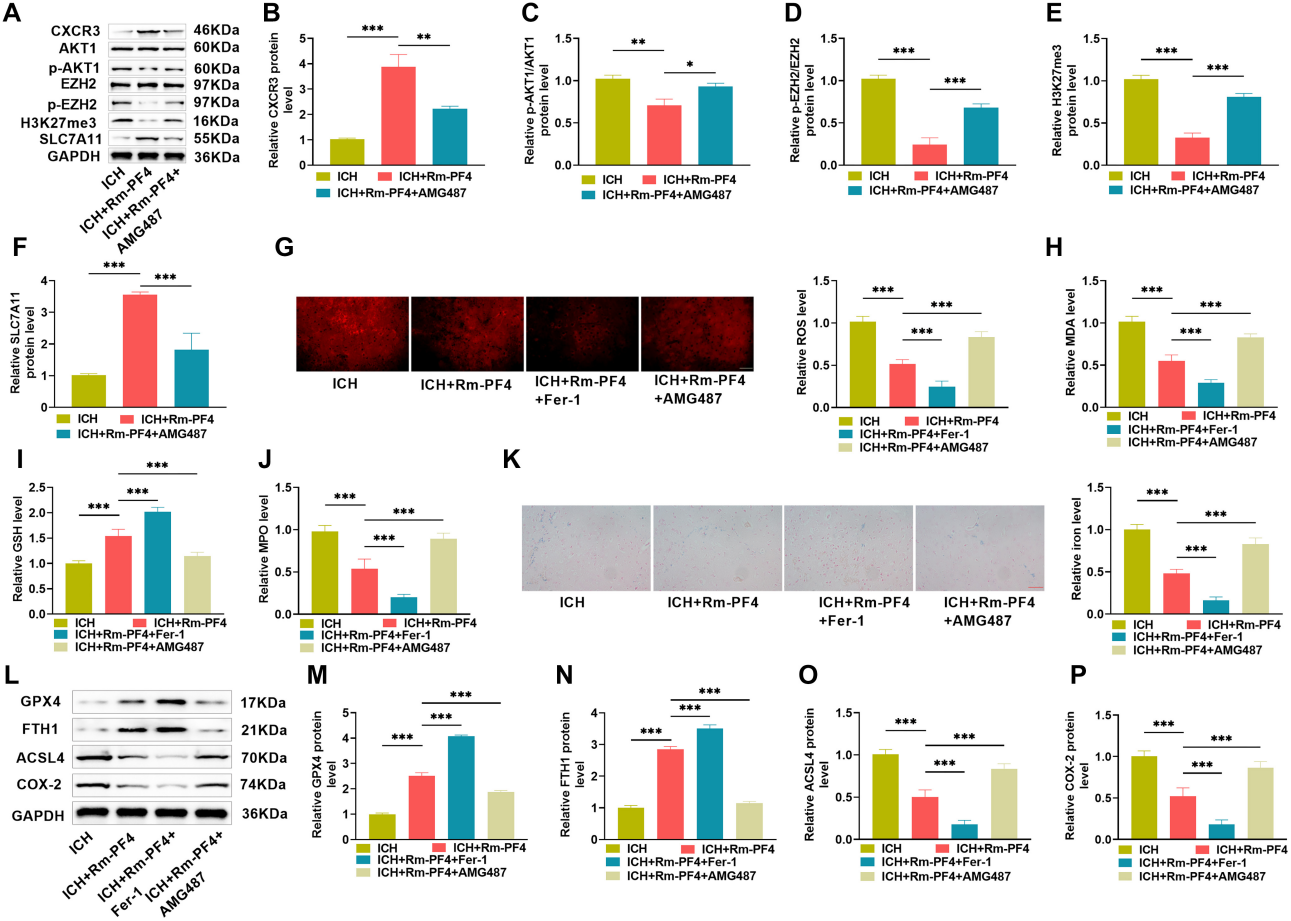
**Rm-PF4 affects the CXCR3/AKT1/SLC7A11 signaling pathway to reduce ferroptosis after ICH.** (A–F) AMG487, a CXCR3 antagonist, was injected into mice brain tissues, and the expression levels of CXCR3/AKT1/SLC7A11 pathway-related proteins were measured by Western blot; (G) DHE kit for measuring ROS levels in mice brain tissues; (H–J) Kits for measuring MDA, GSH, MPO, and iron ion levels in mice brain tissues; (K) Perls staining for iron accumulation in mice brain tissue slices; (L–P) Western blot analysis of ferroptosis-related proteins GPX4, FTH1, ACSL4, and COX-2 in mice brain tissues. Rm-PF4: Recombinant PF4; ICH: Intracerebral hemorrhage; Fer-1: Ferrostatin-1; MPO: Myeloperoxidase; ROS: Reactive oxygen species; MSA: Malondialdehyde.

The above studies have shown that Rm-PF4 reduces PC12 cell damage and the inflammatory response by activating the CXCR3/AKT1/SLC7A11 signaling pathway; hence, we investigated the mechanism of action of Rm-PF4 in attenuating neuroinflammation in vivo. Figure 7A–7F shows that the expression trends of CXCR3/AKT1/SLC7A11 pathway proteins CXCR3, p-AKT1/AKT1, p-EZH2/AKT1, H3K27me3, and SLC7A11 evaluated by western blotting were consistent with the in vitro experimental results. Combined with fluorescent staining and kit assay, as shown in Figure 7G–7J, oxidative stress was attenuated in the brain tissues of the ICH+Rm--PF4 group (decreased levels of ROS, MDA, and MPO, and increased levels of GSH), and the level of oxidative stress was further attenuated by inhibition of ferroptosis by Fer-1 injection, which was reversed after inhibition of the CXCR3/AKT1/SLC7A11 pathway by AMG487 injection, oxidative stress was significantly enhanced (*P* < 0.05). Iron buildup in brain tissue slices was correlated with an increase in oxidative stress (Figure 7K). Similarly, Western blot analysis revealed that Fer-1 increased the levels of ferroptosis-related proteins GPX4 and FTH1 while decreasing the levels of ACSL4 and COX-2, hence strengthening Rm-PF4’s inhibitory action on ferroptosis, which was reversed by AMG487 injection. These data imply that Rm-PF4 prevents ferroptosis following ICH by regulating the CXCR3/AKT1/SLC7A11 signaling pathway.

## Discussion

Platelets, the smallest components of blood, are crucial not only for physiological hemostatic mechanisms but also for maintaining blood vessel wall integrity and facilitating endothelial cell repair [42]. Beyond their traditional role, platelets can function as “inflammatory cells” by promoting neutrophil chemotaxis and activation. PF4, a major constituent of platelet α-granules, is integral to this process [43, 44]. PF4 has been implicated in the pathogenesis of several chronic inflammatory disorders, including atherosclerosis and neurodegenerative diseases [28, 45]. It has been hypothesized that PF4 plays a similar role in the development of ICH. Our findings suggest that platelets, through PF4, mediate hemin-induced ferroptosis, thereby mitigating ICH damage and reducing the associated inflammatory responses. After ICH, heme oxygenase (HO-1) breaks down hemoglobin into iron, carbon monoxide, and biliverdin, releasing substantial amounts of iron into the extracellular space [46]. Iron ions are released into brain tissue 24 h after ICH, and they remain elevated for at least 28 days [47, 48], possibly contributing to perihematoma edema, peroxide buildup, and cell death [49, 50]. Iron excess can directly cause ferroptosis in cells during the pathologic process [51, 52]. Ferroptosis, unlike autophagy, necrosis, apoptosis, and other forms of cell death, is a relatively new type of cell death, characterized by iron accumulation. Ferroptosis is accompanied by morphological, metabolic, and genetic alterations within the cells [53]. Typical hallmarks include alterations in cell shape, mitochondrial shrinkage, outer membrane rupture, iron-dependent buildup of lipid peroxides (e.g., MDA and MPO) and ROS, GSH depletion, decreased GPX4 activity, and iron deposition [54, 55]. When LPO exceeds cellular antioxidant activity, the accumulation of cytotoxic chemicals causes protein breakdown, lipid disruption, and neuronal death, all of which contribute to the pathophysiology of ICH [56, 57]. Furthermore, ferroptosis can be suppressed by specific inhibitors, such as the iron chelator desferrioxamine (DFO) and the LPO inhibitor Fer-1, although it is not susceptible to apoptosis or necrosis inhibitors [58]. In this study, we treated PC12 cells with hemin and the death inducer erastin and found that both had similar inhibitory effects on cell proliferation by CCK-8 and EdU assays. PC12 cells were treated with different inhibitors (apoptosis, cell necrosis, and ferroptosis), and only the ferroptosis inhibitor (Fer-1) acted as previously reported, confirming that hemin could induce ferroptosis in PC12 cells. Fer-1 has been found to alleviate neurological impairments by lowering iron deposition in the brain tissue surrounding hematomas [59], implying that ferroptosis therapies could be a viable therapy method for ICH-induced brain injury. The current study found that treating hemin-induced ICH cell models with PF4 increased cell viability, lowered lipid peroxide and ROS generation, reduced iron deposition, and decreased the levels of inflammatory factors. PF4 injections in ICH animals reduced neurological impairment, lowered ICH volume and edema, increased microglial activation, and blocked inflammatory factor release. Both in vitro and in vivo tests demonstrated that PF4-mediated ferroptosis improved ICH damage.

CXCR3 is a chemokine receptor that PF4 targets [60]. PF4 have demonstrated that studies to bind to the receptor CXCR3, inhibits the inflammatory response, and improves cognitive function during aging [26, 33], whereas SLC7A11 is a critical regulatory protein for ferroptosis, which can prevent ferroptosis [61]. Astrocytes have been shown to interfere with neuronal CXCR3 and inhibit SLC7A11, resulting in neuronal LPO-associated ferroptosis [62], and can prevent neuronal ferroptosis caused by ICH damage by boosting SLC7A11/GPX4 expression [63]. However, the significance of PF4 in controlling ICH-induced neuronal ferroptosis via CXCR3-SLCTA11 remains unknown. It has been demonstrated that histone methylation transferase EZH2 can suppress SLCTA11 production by binding the SLC7A11 promoter via histone H3K27me3 trimethylation modification [34]. It is also known that PF4 can inhibit the phosphorylation of AKT1 by binding to CXCR3 [64, 65] and that active AKTl can activate EZH2 [66]. As a result, we hypothesized that PF4 binds to and inhibits the receptor CXCR3, inhibits the activation of AKT1 and EZH2, reduces trimethylation of the SLC7A11 promoter H3K27me3, and increases SLC7A11 expression, which in turn mitigates LPO of neuronal cells, reduces ferroptosis, and attenuates secondary injury after ICH. Western blot analysis revealed that the application of a CXCR3 antagonist affected PF4’s control of the CXCR3/AKT1/SLC7A11 pathway proteins in ICH cells, resulting in the downregulation of CXCR3 and SLC7A11 proteins and upregulation of phosphorylated AKT1, EZH2, and H3K27me3 proteins. A similar experiment was performed using the ICH mouse model. These results confirm that PF4 inhibits ferroptosis-induced neuroinflammation following ICH via the CXCR3/AKT1/SLC7A11 signaling pathway.

In summary, PF4 can regulate the accumulation of iron ions and increase oxidative stress in brain tissue cells via the CXCR3/AKT1/SLC7A11 axis, inhibit the production of LPO products, and prevent neurons from undergoing ferroptosis, thereby alleviating ICH injury in mice (Figure S1). More emphasis should be placed on aggressive antiplatelet therapy for ICH, such as targeting PF4 to inhibit platelet aggregation and inflammatory chemotaxis and alleviate the secondary injury of ICH, thus improving the prognostic outcome of ICH patients. In this study, neuronal function models were prepared using PC12 cells, but they were derived from rat adrenal pheochromocytoma rather than from primary neurons. Thus, PC12 cells have application limitations, and further consideration needs to be given to validate the findings in primary neuronal cultures in the future to strengthen the relevance of this study to neuronal iron prolapse. In addition, this content needs to be further deepened and expanded. Animal models and in vitro systems may not be able to fully mimic human disease conditions, and more clinical studies need to be conducted to verify their feasibility.

## Conclusion

This study is the first to investigate how PF4 regulates ferroptosis in an ICH model, hypothesizing that the CXCR3/AKT1/SLC7A11 signaling pathway mediates PF4’s action. The research content is of great innovative value, as it can fill an important gap in the knowledge related to neuronal cell death after ICH and provide an important theoretical basis and therapeutic target for translational research on the clinical treatment of ICH. In the future, we should expand our research on the effects of PF4 on cell autophagy, necrosis, apoptosis, and its mechanism after ICH, and continue to dig deeper into the mechanistic proteins and pathways of PF4, explore the interaction targets between different cell death programs, and form a complete network of regulated cell death mechanisms, which is of great significance for guiding the development of PF4 pharmacological agents and exerting an overall regulation. Meanwhile, it should be investigated whether several signaling pathways are active concurrently during PF4 inhibition of ICH ferroptosis, which leads to the altered operation of cellular regulatory systems. Therefore, additional in-depth research and convincing experimental and clinical evidence are required to further develop ICH therapies in the future.

## Supplemental data

**Figure S1.**
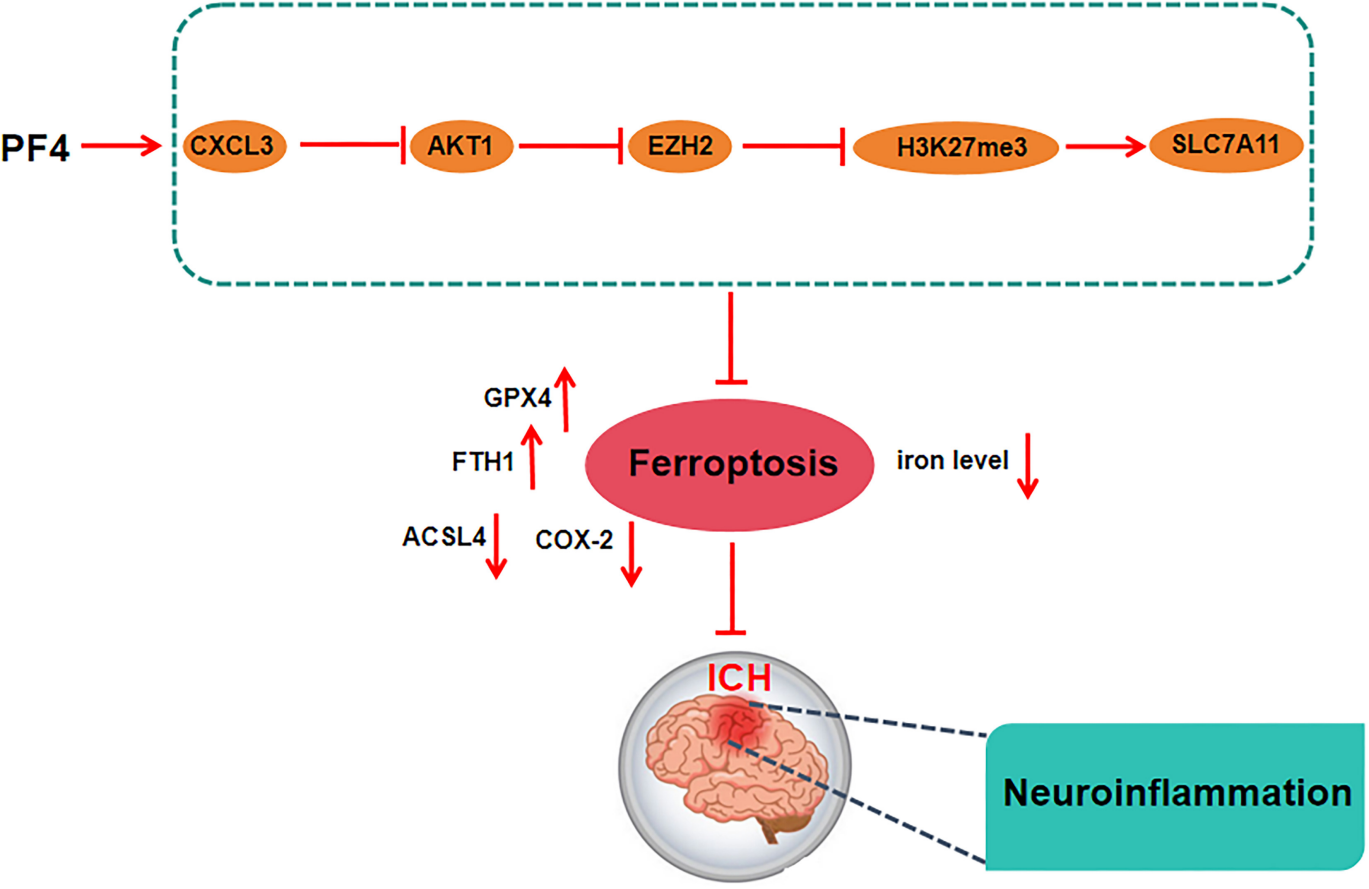
**Graphical abstract.** PF4 regulates the expression of ferroptosis-related proteins (upregulation of GPX4 and FTH1, downregulation of ACSL4 and COX-2) by activating the CXCR3/AKT1/SLC7A11 pathway, which reduces iron deposition and prevents ferroptosis in neurons, thereby reducing ICH injury in mice. ICH: Intracerebral hemorrhage; PF4: Platelet factor 4.

## Data Availability

The data supporting the findings of this study can be obtained from the corresponding author, upon request.
